# Place of Preoperative Treatment of Acromegaly with Somatostatin Analog on Surgical Outcome: A Systematic Review and Meta-Analysis

**DOI:** 10.1371/journal.pone.0061523

**Published:** 2013-04-25

**Authors:** Francisco Pita-Gutierrez, Sonia Pertega-Diaz, Salvador Pita-Fernandez, Lara Pena, Gloria Lugo, Susana Sangiao-Alvarellos, Fernando Cordido

**Affiliations:** 1 Department of Endocrinology, University Hospital A Coruña, A Coruña, Spain; 2 Clinical Epidemiology and Biostatistics Unit, University Hospital A Coruña, A Coruña, Spain; 3 Department of Investigation, University Hospital A Coruña, A Coruña, Spain; 4 Department of Medicine, University of A Coruña, A Coruña, Spain; University of Cordoba, Spain

## Abstract

**Context:**

Transsphenoidal neurosurgery is the accepted first-line treatment of acromegaly in the majority of patients. Previous studies addressing preoperative somatostatin analog (SSA) treatment and subsequent surgical cure rates are conflicting, reporting either benefits or no significant differences.

**Objective:**

The aim of this study, based on a meta-analysis of all published reports, was to investigate whether treatment with SSA before surgery improves the surgical outcome of acromegaly.

**Data Sources:**

All studies of preoperative treatment of acromegaly with SSA were systematically reviewed up to December 2011. We searched the Medline, Embase, Cochrane and Google Scholar electronic databases. Study Selection: The primary endpoint was the biochemical postoperative cure rate. We identified 286 studies, out of which 10 studies (3.49%) fulfilling the eligibility criteria were selected for analysis; five retrospective studies with a control group, two prospective non-randomized trials, and three prospective controlled trials. The meta-analysis was conducted using the random-effects model.

**Data Extraction:**

Data were extracted from published reports by two independent observers. Data Synthesis: A borderline effect was detected in the analysis of all of the trials with control groups, with a pooled odds ratio (OR) for biochemical cure with SSA treatment of 1.62 (95% CI, 0.93–2.82). In the analysis of the three prospective controlled trials, a statistically significant effect was idenfified OR: 3.62 (95% CI, 1.88–6.96).

**Conclusions:**

Preoperative treatment with SSA og GH-secreting pituitary adenomas shows a significant improvement on surgical results. This meta-analysis suggests that in centers without optimal results all patients with a GH-secreting pituitary macroadenoma should be treated with a long-acting SSA prior to surgical treatment.

## Introduction

Acromegaly is a rare but severe endocrine disease due to excess GH production, caused by a pituitary adenoma in the vast majority of cases (around 98%). The incidence of acromegaly is approximately 5 cases per million per year and the prevalence is 60 cases per million [1]. The diagnosis is often preceded by around 10 years of active but unrecognized disease [2]. Somatotroph adenomas are monoclonal in origin and develop as a result of genetic changes. Over 90% of patients with acromegaly have benign monoclonal pituitary adenomas, which are not surrounded by hyperplasic tissue [3]. Other contributors that could facilitate the expansion of the somatotroph tumor cells are hypothalamic GHRH or somatostatin, paracrine growth factors and ghrelin [2], [4]. Recent data suggest the importance of the role of ghrelin in the pathophysiological regulation of GH [5]–[7]. Current treatment for acromegaly includes neurosurgery, radiotherapy and medical therapy with dopamine agonists, somatostatin analog (SSA), and the GH-receptor antagonist pegvisomant [2], [8]–[10].

Transsphenoidal neurosurgery is the accepted first-line treatment of acromegaly in the majority of patients. Outcome predictors include tumour size, extrasellar extension, cavernous sinus or dural invasion, pretreatment GH and IGF-I levels and surgical expertise [11], [12]. The best reported cure rates for microadenomas and macroadenomas are 80–90% and 50–60% respectively [11]–[14]. In the Belgian registry of acromegaly, a survey of “real life” outcome in 418 acromegalic subjects, the cure rate of surgery by the definition of both normal IGF for age and GH <2 µg/l was 34% [15]. Overall cure rates as low as 18% (39% microadenomas and 12% macroadenomas) have been reported [16]. It is possible that other studies with low cure rates remain unpublished.

Primary or secondary medical treatment of acromegaly with somatostatin analog (SSA) can lead to relief of symptoms [17] and normalized GH and IGF-I [18]. SSA treatment may cause shrinkage of GH-secreting pituitary adenomas [18], [19]. Theoretically, this could improve the likelihood of a radical resection. Furthermore, it has been suggested that SSA treatment softens the tumor parenchyma and thereby facilitates tumor removal [20], [21]. It has also been reported that SSA pretreatment leads to a shortening of postoperative hospital stay [22].

Previous studies addressing preoperative SSA treatment and subsequent surgical cure rates are conflicting, reporting benefit [21]–[24] or no difference with preoperative SSA treatment [20], [25]–[29]. In recent guidelines, a role for presurgical medical therapy with SSA to improve biochemical outcomes with surgery has been suggested, although further studies are needed to support general use [30]. Moreover, there is currently insufficient evidence to recommend it for improved surgical outcome or postoperative complications [31].

The aim of this study, based on meta-analysis of all published reports, was to investigate whether treatment with somatostatin analogs before surgery improves the surgical outcome of acromegaly.

## Materials and Methods

### Identification of Relevant Articles

All studies on the preoperative treatment of acromegaly with somatostatin analogs were systematically reviewed up to December 2011 (key words: acromegaly AND preoperative AND somatostatin analogs AND (octreotide OR lanreotide). The search strategy was unrestricted. We included studies investigating the effect of preoperative somatostatin analogs treatment on postoperative cure rates, searching the Medline, Embase, Cochrane and Google Scholar electronic databases. With the aim of identifying additional candidate studies, we reviewed the reference list of the eligible primary studies, narrative reviews and systematic reviews. In cases where multiple publications existed from the same study, the article with the most information was included. We identified 286 studies; we then exclude studies that were not original, studies that did not report outcomes of interest, studies whose full text could not be obtained, and studies with insufficient data for meta-analysis. We also excluded studies that did not report the surgical outcome or measure IGF-I. We identified twenty-one studies: eleven prospective or retrospective studies without a control group [32]–[42], five retrospective studies with a control group [20]–[22], [27], [28], two non-randomized prospective studies with control group [25], [26] and three randomized prospective controlled trials [23], [24], [29]. A summary of the follow-up strategy used to identify the studies is shown in Figure S1. The studies without a control group were excluded from meta-analysis, as a result of which only 10 studies were finally selected (Table S1).

### Data Extraction

Data were extracted from published reports by two independent observers (FPG and FC). Discrepancies were resolved by discussion among the authors of this report. The following data were extracted: description of study characteristics, age, gender distribution, number of patients included, postoperative biochemical remission criteria as defined in the different studies (main objective, age adjusted normal IGF-I, fasting GH and GH after oral glucose tolerance test, Table S1), type and dose of somatostatin analog, preoperative medical treatment duration, time of postoperative evaluation, adenoma size (micro-or macroadenoma), postoperative complications, the effect of treatment on tumor size. The studies were generally of good quality, with few losses to follow-up and the use of appropriate statistical methods.

### Statistical Analysis

The results are expressed as the mean (SD), median and range, absolute values and percentage. The main outcome of interest was the percentage of postoperative biochemical cure rate. The odds ratio (OR) was used as a measure of association, with its 95% confidence interval. Statistical heterogeneity was assessed using Galbraith and L’Abbé plots, and the Q statistic from DerSimonian and Laird. The meta-analysis was conducted using the random-effects model, because of the marked clinical heterogeneity. The possibility of publication bias was assessed using a funnel plot. Funnel plot asymmetry was evaluated by Begg’s and Egger’s tests, and a significant publication bias was considered if the *P* value was <0.05. A sensitivity analysis was also carried out by evaluating the influence of the absence of each individual study on the global measure of association. Furthermore, a subgroup analysis was performed including the three randomized prospective controlled trials.

Data were analyzed by EPIDAT 3.1 software (Xunta de Galicia/PHO, A Coruña, Galicia, Spain, 2006). All reported p-values are two sided, with significance set at p<0.05.

## Results

Meta-analysis was first performed using all of the studies with a control group (n = 10) and then with randomized prospective controlled trials (n = 3).

### All Identified Trials with Control Group (n = 10)

The characteristics of the retrospective or prospective studies with a control group (n = 10) are shown in table 1. Participants in the trials tend to be middle-aged men or women; the mean age range was 40.6–47.5 years, with a percentage range for females of 40–54%. The number of patients in each trial ranged from 24 in the study by Kristoff et al [25] to 286 in the study by Losa et al [28].

The type of SSA used in most of the studies was short acting octreotide (table S1). In the most recent studies-Shen et al [29] and the Carlsen et al trial [23]-octreotide LAR was used, 20 mg im every 28th day for 3 months and 20 mg im every 28th day for 6 months, respectively. In the Losa et al study [28] several SSA were utilized, and in the study of Mao et al [24] lanreotide SL was used, starting with 30 mg/2 weeks im and increasing to 30 mg/week im at week 8 if mean GH >2.5 µg/l on GH day curves. The duration of preoperative treatment was variable, with a mean range between 5.8 to 36 weeks. The postsurgical evaluation time varied from 1 week (with short acting SSA) to more than 24 weeks. Several methodological problems were identified: five of the studies [20]–[22], [27], [28] were retrospective, with short acting octreotide. The study by Biersmaz et al [26] was a prospective non-randomized study with a reduced sample size. On the other hand, the SSA was short acting and pretreatment IGF-I values were higher in the treated group than in the control group.

Differences in cure rates between treatment groups in the included studies are detailed in table S2. The cure rate was higher in the treatment group in the studies by Stevenaert et al [21], Colao et al [22], Plockinger et al [27], Carlsen et al [23], Mao et al [24] and Shen et al [29]. A statistically significant effect was detected in the studies of Stevenaert et al [21] and Mao et al. [24]. Figure S2a shows the effect of SSA pretreatment on biochemical cure rate in all of the trials with a control group. The treatment shows a borderline significant effect, with a pooled OR (random effects) for biochemical cure of 1.62 (95% CI, 0.93–2.82). Similar results were obtained after a sensitivity analysis was performed (Table S3). This analysis determines the overall effect (global OR) after removal of the meta-analysis of each of the individual studies. This analysis shows that the probability of cure is higher in the treatment group than in the control group, even though no statistical significance was achieved.

Surgical outcome in published studies varies greatly. We analyzed if there was a relationship between untreated surgical outcome and SSA pretreatment results. Regression analysis of the cured percentage in untreated patients versus the odds ratio of the ten studies analyzed and presented in table S2, revealed a highly significant linear relationship (Spearman Rho = −0.842, adjusted R2 = 0.806, P<0.0001) indicating that centers with good surgical results do not benefit from pretreatment and centers with worse surgical results benefit most from pretreatment. (Figure S3).

### Tumor Shrinkage, Hospital Stay and Surgical Complications

The mean volume reduction in the treated group was evaluated in several studies. It varied from 40% in the study of Kristoff et al [25] to 25% in the trial of Plockinger et al [27]. Colao et al [22] found a shorter hospital stay in the treated group versus control group (5.6±0.5 vs 8.6±0.7 days), whereas Biersmaz et al [26] found no difference (8.2±8 vs 7.1±7). The surgical complications evaluated prospectively in the studies by Carlsen et al [23] and Mao et al [24] found no difference between treated and control groups.

### Randomized Prospective Controlled Trials (n = 3)

When only the three randomized prospective controlled trials [23], [24], [29] were analyzed, the following results were found. The differences in cure rates between treatment groups in the prospective trials are detailed in figure S2b. The treatment effect was significant, with a pooled OR (random effects) for biochemical cure with SSA treatment of 3.62 (95% CI, 1.88–6.96).

### Publication Bias

We examined the funnel plot for signs of publication bias (Figure S4). No asymmetry or small-study effects were detected, and the Begg’s and Egger’s tests were not statistically significant (P>0.05).

## Discussion

This systematic review and meta-analysis refines the place of treatment with somatostatin analog before surgery in the surgical outcome of acromegaly. Based on biochemical cure and only including prospective randomized trials, preoperative treatment with somatostatin analog of GH-secreting pituitary adenomas shows a significant improvement on the surgical results, with an odds ratio (random effects) of 3.62 (95% CI, 1.88–6.96). We have found a highly significant relationship between untreated surgical outcome and SSA pretreatment results. These data indicate that centers with good surgical results do not benefit from pretreatment and centers with worse surgical results benefit most from pretreatment.

Transsphenoidal neurosurgery is the accepted first-line treatment of acromegaly in the majority of patients. Even if it is not curative, surgical debulking of pituitary macroadenomas causing acromegaly improves control by SSA [43], [44]. The outcome of treatment with somatostatin analogs as primary or secondary therapy in clinical practice has been recently reviewed by Colao et al [8], controlling excess GH in 60–62% of patients with normalization of IGF-I levels in 59–49% of patients. Tumor shrinkage, evaluated as a reduction of at least 20% compared to the baseline, was observed in 56–75% of patients treated with somatostatin analogs. SSA efficacy is high, and only around 25% of the patients have a poor response or resistance, when considering biochemical and tumoral effects jointly [8]. A recent meta-analysis on the effects of octreotide on tumor mass in acromegaly has found that octreotide LAR induces clinically relevant tumor shrinkage in more than half of patients with acromegaly, and that intramuscular octreotide LAR produced tumor shrinkage in twice as many patients as subcutaneous octreotide [45]. These data suggest a possible explanation for the improved results with long-acting SSA when compared to studies with subcutaneous octreotide. The results of the present study are mainly based on prospective studies in patients with macroadenoma [23], [24], [29], and should probably not be applied to microadenoma patients. In the study by Carlsen et al [23], presurgical treatment did not improve surgical results in microadenoma patients. The SSA doses employed in the three prospective controlled trials were for octreotide LAR 20 mg im every 28th day and for lanreotide SL up to 30 mg/week im.Theoretically it could be possible that higher SSA doses improve the surgical outcome, although there are no studies analyzing SSA pretreatment with different doses. There are data indicating that high SSA doses could increase its affinity for somatostatin receptor 5 or induce an up regulation of somatostatin receptor 2 or alter its degradation [46].

There are some clinical data which suggest that the main reason for the improved surgical outcome in the treated group was that drug pretreatment made some of the tumors less invasive [29]. However, other mechanisms that could modify the biology of the GH-secreting pituitary tumor cannot be excluded; recent studies have found that somatostatin analogs pretreatment modulate aryl hydrocarbon receptor interacting protein in somatotroph adenomas [47]. We believe that the present results are very important for the treatment of acromegaly, in real clinical practice. Outside of international reference centers, the surgical cure of acromegaly is low [15], [16] or largely unknown; any clinical intervention capable of improving surgical results should be greatly appreciated. Regression analysis of the cured percentage in untreated patients versus the odds ratio of the pretreatment effect on the ten studies analyzed, revealed a highly significant linear relationship (Spearman Rho = −0.842, adjusted R2 = 0.806, P<0.0001).

When we compare the outcome of the preoperative treatment in the prospective randomized studies with the retrospective studies, the results are clearly better in the former. These results could be due to the fact that, from a clinical practice perspective, there should probably be a tendency to treat preoperatively patients with more biochemically active acromegaly, with greater GH and IGF-I. Patients with more biochemically active acromegaly have a worse prognosis for postoperative cure [8], [11], [12]. Moreover, in some prospective studies with a control group, the treated group had a greater IGF-I than the control group [26].

We analyzed the possibility of publication bias (when only the positive results are published) using a funnel plot (Figure S4). No asymmetry or small-study effects were detected, suggesting the absence of publication bias.

We could not conduct a specific meta-analysis for adenomas considered to be non-resectable due to a lack of information in most of the articles reviewed. Some studies suggest that presurgical treatment did not improve the results in macroadenomas considered to be non-resectable [20]. We lack sufficient data to analyze the length of hospital stay or surgical complication rates. The most recent prospective randomized trials did not observe any difference in hospital stay or surgical complication rates between preoperative SSA treatment or not [23], [24], [29].

This study has several limitations. The first is the small number of studies available. To overcome this limitation, we decided to pool the prospective and retrospective studies. In this analysis, although there was a clear trend towards an improvement in the cure rate, the results show a positive borderline significant effect. However, when we only analyzed prospective randomized control studies (n = 3), we found a clearly significant improvement in the surgical cure with SSA pretreatment. The second limitation is the length of the postoperative evaluation period. An ideal period could be 1 year after SSA withdrawal, in order to exclude any lingering effect of presurgical SSA treatment on the outcome [48]. However, when considering the prospective randomized trials, the evaluation in the study by Carlsen et al [23] was performed 3 months postoperatively, and in the study by Mao et al and Shen et al [24], [29] at least 4 months postoperatively, both intervals considered as a “safe” period to allow for washout of SSA effects [48], [49].

In conclusion, the present meta-analysis suggests that in centers without optimal surgical results all patients with a GH-secreting pituitary macroadenoma should be treated with a long-acting somatostatin analog prior to surgical treatment.

## Supporting Information

Figure S1 **Preoperative treatment of acromegaly with somatostatin analog on surgical outcome.** Literature review.
(TIF)

Figure S2 **Forest plot of trials of preoperative treatment of acromegaly with somatostatin analog on surgical outcome.** Figure S2a: All identified trials with control group (n = 10). Figure S2b: Randomized Prospective controlled trials (n = 4).
(TIF)

Figure S3 **Regression analysis: Cured percentage in untreated patients versus the Odds Ratio (from Table S2).**
(TIF)

Figure S4 **Funnel plot of preoperative treatment of acromegaly with somatostatin analog on surgical outcome.**
(TIF)

Table S1 **Characteristics of the studies, with control group, evaluating the effect of preoperative treatment of acromegaly with somatostatin analog (SSA) on surgical outcome.**
(DOC)

Table S2 **Preoperative treatment of acromegaly with somatostatin analog on surgical outcome.** Differences in cure rates between treatment groups in the studies included.
(DOC)

Table S3 **Preoperative treatment of acromegaly with somatostatin analog on surgical outcome.** Sensitivity analysis.
(DOC)
