# Supplementary figures and images for: Place of Preoperative Treatment of Acromegaly with Somatostatin Analog on Surgical Outcome: A Systematic Review and Meta-Analysis
Source: PLoS One. 2013 Apr 25;8(4):e61523. doi: 10.1371/journal.pone.0061523 (PMC3636268; doi:10.1371/journal.pone.0061523)

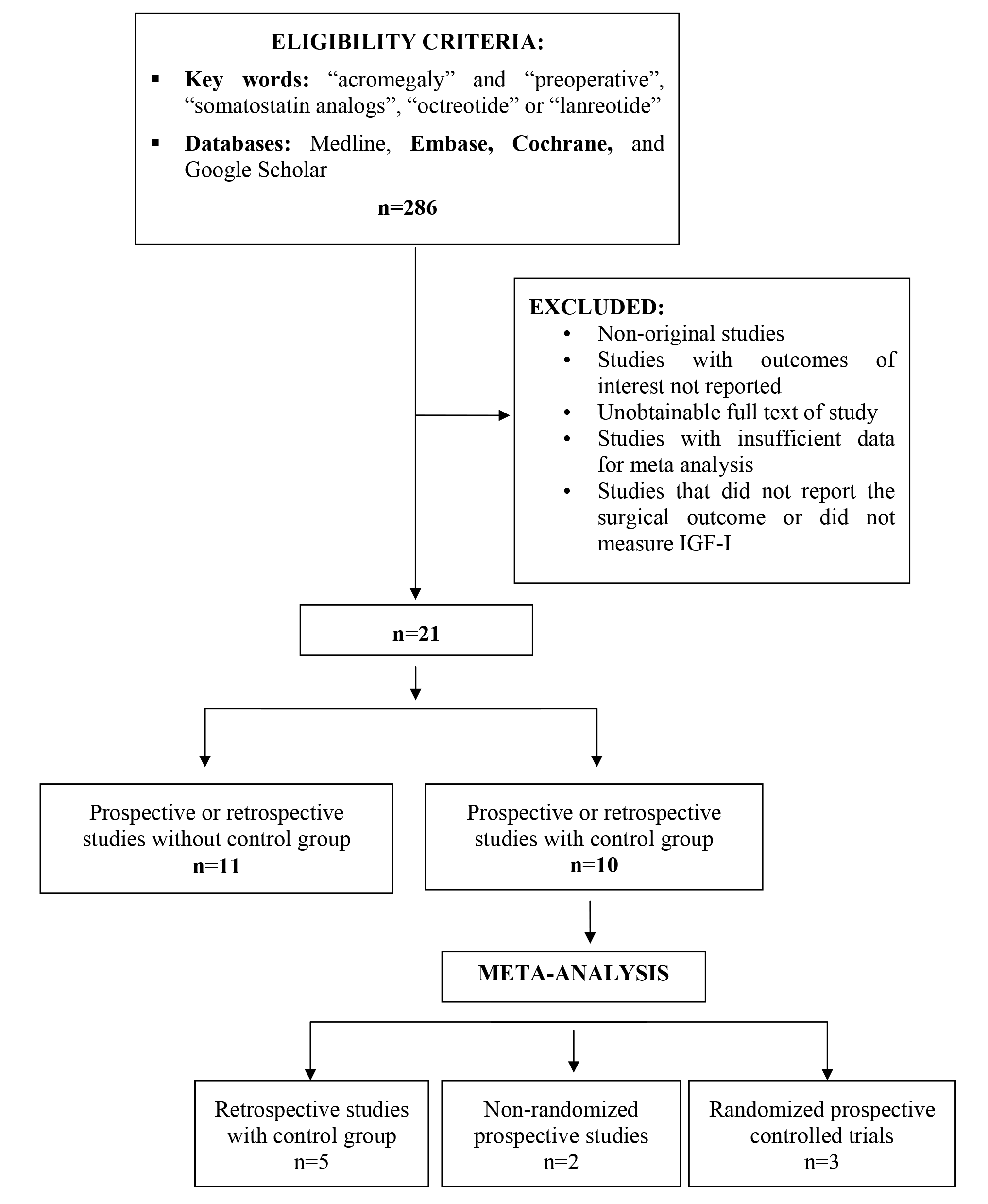

Supplement: Figure S1 — Preoperative treatment of acromegaly with somatostatin analog on surgical outcome. Literature review. (TIF) [file pone.0061523.s001.tif]

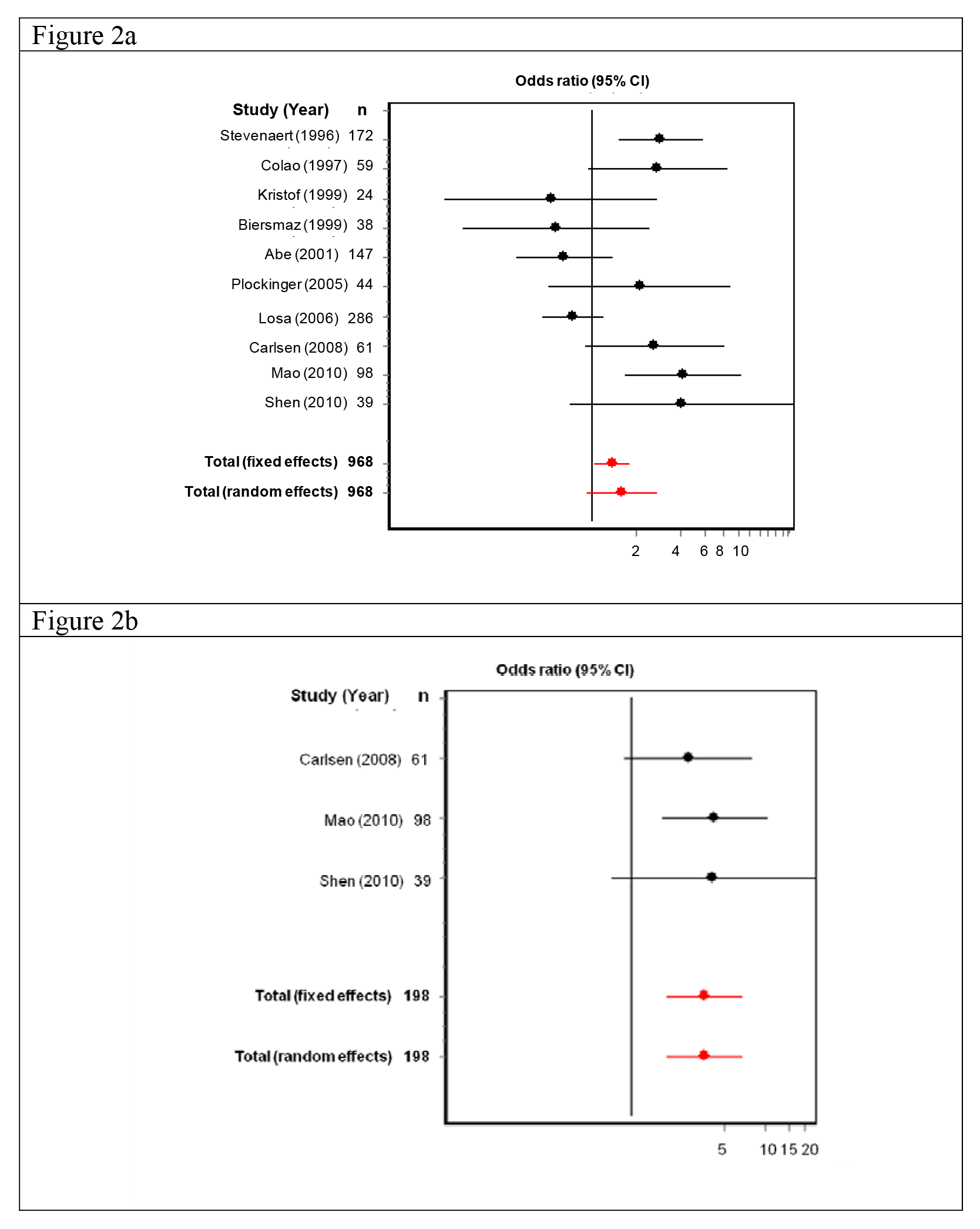

Supplement: Figure S2 — Forest plot of trials of preoperative treatment of acromegaly with somatostatin analog on surgical outcome. Figure S2a: All identified trials with control group (n = 10). Figure S2b: Randomized Prospective controlled trials (n = 4). (TIF) [file pone.0061523.s002.tif]

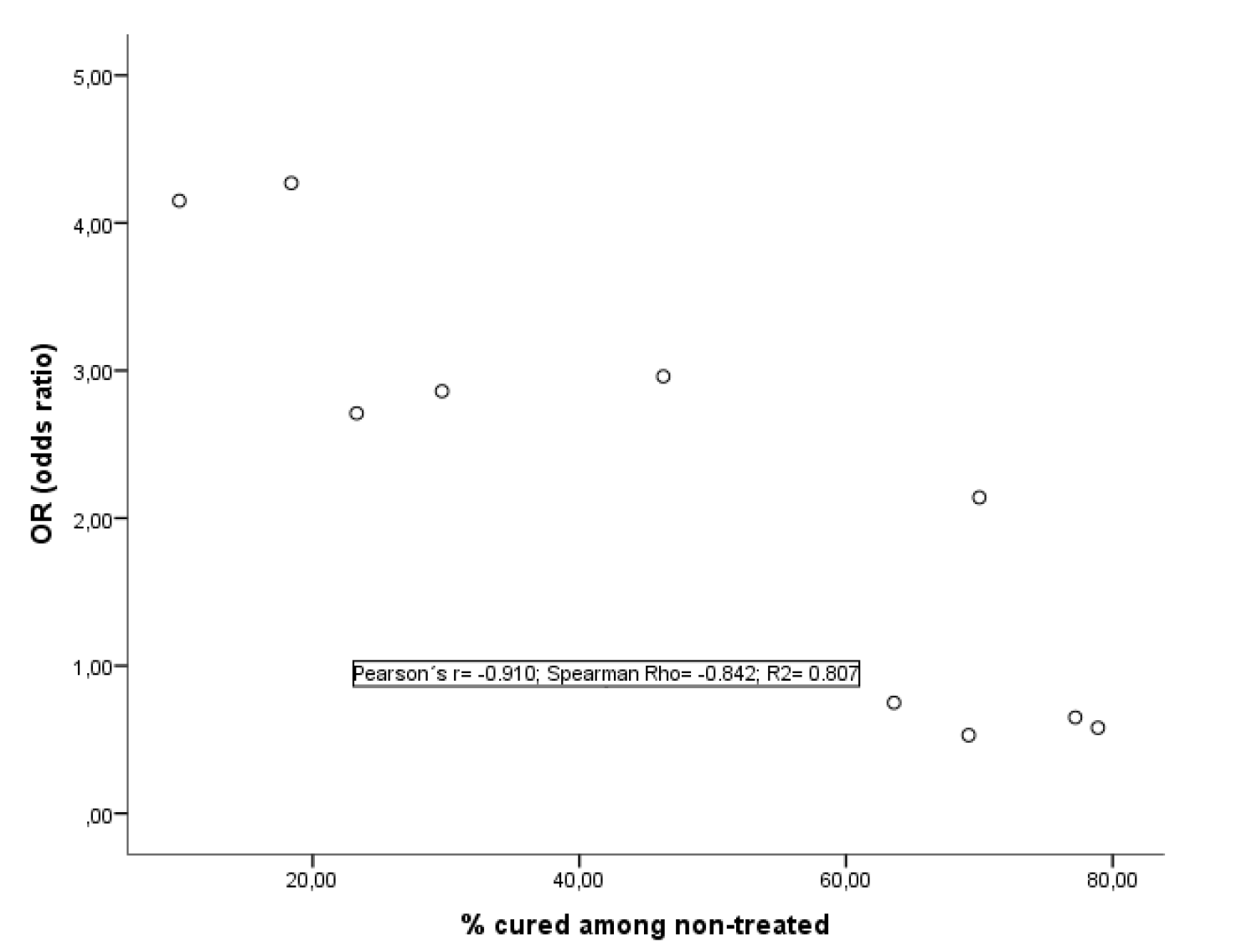

Supplement: Figure S3 — Regression analysis: Cured percentage in untreated patients versus the Odds Ratio (from Table S2). (TIF) [file pone.0061523.s003.tif]

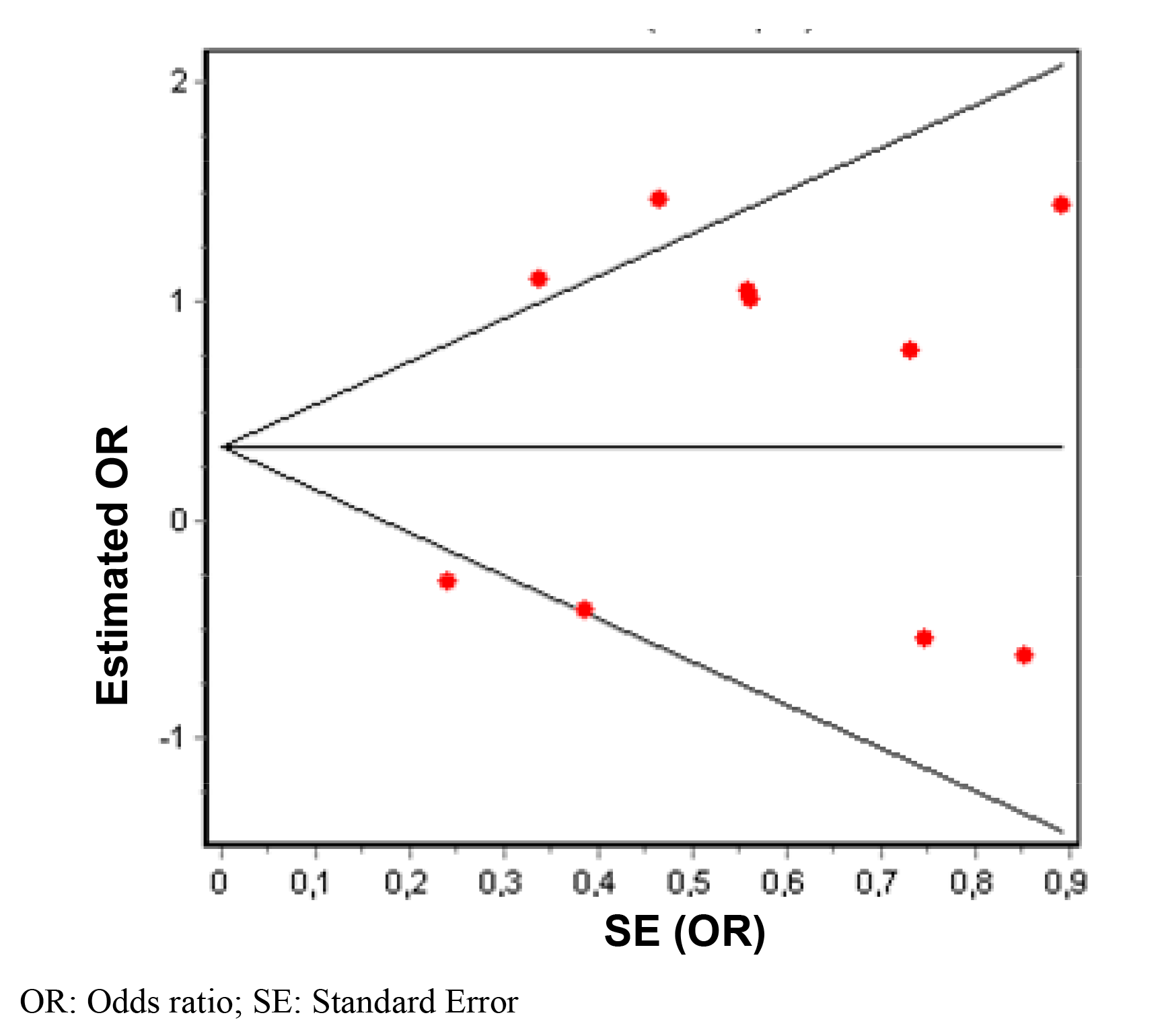

Supplement: Figure S4 — Funnel plot of preoperative treatment of acromegaly with somatostatin analog on surgical outcome. (TIF) [file pone.0061523.s004.tif]
