# Supplementary material for: Place of Preoperative Treatment of Acromegaly with Somatostatin Analog on Surgical Outcome: A Systematic Review and Meta-Analysis
Source: PLoS One. 2013 Apr 25;8(4):e61523. doi: 10.1371/journal.pone.0061523 (PMC3636268; doi:10.1371/journal.pone.0061523)
Supplement: Table S1 — Characteristics of the studies, with control group, evaluating the effect of preoperative treatment of acromegaly with somatostatin analog (SSA) on surgical outcome. (DOC) [file pone.0061523.s005.doc]

Table S1.

| First author (year) | Design of the study | No. of patients: treated / non-treated | Mean age (yr) | Percentage of females (%) | Type of SSA dose and treatment duration (weeks) | Cure rate criteria | Postsurgical evaluation time (weeks) | Study  Weaknesses |
| --- | --- | --- | --- | --- | --- | --- | --- | --- |
| Stevenaert (1996) | Retrospective | 64/108 | 42.5 | 54 | Octreotide 100 µg/8h (3-16) | IGF I  OGTT<1  GH<2 | >24 | Retrospective,  short acting analog |
| Colao (1997) | Retrospective | 22/37 | 40.6 | 54 | Octreotide 100 µg/8h (12-24) | IGF I  OGTT<2 | 1-2/48 | Retrospective,  short acting analog |
| Kristof (1999) | Prospective | 11/13 | 43.0 | 42 | Octreotide 100 µg/8h (16.5±10) | IGF I  OGTT<1 | 12 | Not random  Small group,  short acting analog |
| Biersmaz (1999) | Prospective | 19/19 | 43.5 | 42 | Octreotide 100 µg/8h (5.8±0.8) | IGF I  OGTT<1  GH<5 | >24 | Not random,  patient election,  IGF-I higher in treated |
| Abe  (2001) | Retrospective | 90/57 | 46.9 | 49 | Octreotide 100 µg/8h (36±5.6) | IGF I  GH < 2.5 | >24 | Retrospective,  short acting analog |
| Plockinger (2005) | Retrospective | 24/20 | 45.5 | 50 | Octreotide 100 µg/8h (12-24) | IGF I  OGTT<1 | >24 | Retrospective, not random,  short acting analog |
| Losa (2006) | Retrospective | 143/143 | 44.5 | 53 | Several SSA (>12) | IGF I  OGTT<1 | 16-24 | Retrospective |
| Carlsen (2008) | Prospective | 31/30 | 47.5 | 50 | Octreotide LAR  20 mg/28 days (24) | IGF I | 12 |  |
| Mao (2010) | Prospective | 49/49 | 46.2 | 40 | Lanreotide SL  30 mg/14 days (12) | IGF I | 16 |  |
| Shen (2010) | Prospective | 19/20 | 41.7 | 54 | Octreotide LAR  20 mg/28 days (12) | IGF I  OGTT<1 | >24 |  |
