# Supplementary material for: Place of Preoperative Treatment of Acromegaly with Somatostatin Analog on Surgical Outcome: A Systematic Review and Meta-Analysis
Source: PLoS One. 2013 Apr 25;8(4):e61523. doi: 10.1371/journal.pone.0061523 (PMC3636268; doi:10.1371/journal.pone.0061523)
Supplement: Table S2 — Preoperative treatment of acromegaly with somatostatin analog on surgical outcome. Differences in cure rates between treatment groups in the studies included. (DOC) [file pone.0061523.s006.doc]

Table S2.

| Author (year) | No. treated /  No. non-treated | % cured  among treated | % cured  among  non-treated | Difference | OR | 95% CI |
| --- | --- | --- | --- | --- | --- | --- |
| Stevenaert (1996) | 64/108 | 71.9% | 46.3% | 25.6% | 2.96 | 1.52-5.75 |
| Colao (1997) | 22/37 | 54.5% | 29.7% | 24.8% | 2.84 | 0.95-8.49 |
| Kristof (1999) | 11/13 | 54.5% | 69.2% | -14.7% | 0.53 | 0.10-2.84 |
| Biersmaz (1999) | 19/19 | 68.4% | 78.9% | -10.5% | 0.58 | 0.13-2.51 |
| Abe (2001) | 90/57 | 68.9% | 77.2% | -8.3% | 0.65 | 0.31-1.40 |
| Plockinger (2005) | 24/20 | 83.3% | 70.0% | 13.3% | 2.14 | 0.51-9.02 |
| Losa (2006) | 143/143 | 56.6% | 63.6% | -7.0% | 0.75 | 0.46-1.20 |
| Carlsen (2008) | 31/30 | 45.2% | 23.3% | 21.8% | 2.71 | 0.90-8.15 |
| Mao (2010) | 49/49 | 49.0% | 18.4% | 30.6% | 4.27 | 1.71-10.65 |
| Shen (2010) | 19/20 | 31.6% | 10% | 21.6% | 4.15 | 0.72-23.95 |
| *OR: Odds Ratio; CI: Confidence Interval* | | | | | | |
