# Supplementary material for: Place of Preoperative Treatment of Acromegaly with Somatostatin Analog on Surgical Outcome: A Systematic Review and Meta-Analysis
Source: PLoS One. 2013 Apr 25;8(4):e61523. doi: 10.1371/journal.pone.0061523 (PMC3636268; doi:10.1371/journal.pone.0061523)
Supplement: Table S3 — Preoperative treatment of acromegaly with somatostatin analog on surgical outcome. Sensitivity analysis. (DOC) [file pone.0061523.s007.doc]

Table S3.

| Excluded study  (Author (year)) | No. patients in meta-analysis | Global OR  (Random effects) | 95% CI (OR) | %change in global OR |
| --- | --- | --- | --- | --- |
| Stevenaert (1996) | 757 | 1.36 | 0.74-2.48 | -10.5% |
| Colao (1997) | 870 | 1.41 | 0.76-2.62 | -7.2% |
| Kristof (1999) | 905 | 1.65 | 0.90-3.00 | 8.3% |
| Biersmaz (1999) | 891 | 1.66 | 0.90-3.04 | 9.0% |
| Abe (2001) | 782 | 1.73 | 0.93-3.22 | 13.8% |
| Plockinger (2005) | 885 | 1.47 | 0.79-2.73 | -3.2% |
| Losa (2006) | 643 | 1.74 | 0.96-3.16 | 14.7% |
| Carlsen (2008) | 868 | 1.42 | 0.76-2.64 | -6.7% |
| Mao (2010) | 831 | 1.32 | 0.74-2.33 | -13.3% |
| Shen (2010) | 929 | 1,52 | 0,86-2,70 | -6,1% |
| *OR: Odds Ratio; CI: Confidence Interval* | | | | |
